# The Mechanism and Potential Therapeutic Strategies of Vascular Aging

**DOI:** 10.1002/agm2.70043

**Published:** 2025-10-10

**Authors:** Wan‐Zhou Wu, Xuan Wang, Fang‐Fang Wu, Xiao‐Tian Yan, Yong‐Ping Bai

**Affiliations:** ^1^ National Clinical Research Center for Geriatric Disorders, Xiangya Hospital Central South University Changsha Hunan China; ^2^ Department of Geriatric Medicine, Xiangya Hospital Central South University Changsha Hunan China; ^3^ Department of Pediatric Loudi Central Hospital Loudi Hunan China; ^4^ Department of Pediatric The First Hospital of Changsha Changsha Hunan China; ^5^ Department of Cardiology Medicine, Xiangya Hospital Central South University Changsha Hunan China

**Keywords:** endothelial dysfunction, therapy, vascular aging, vascular stiffness

## Abstract

As we approach the mid‐21st century, more developing countries are progressing towards becoming developed nations. Advances in medical technology have resulted in the prolonged human lifespan and a sustained decline in birth rates, leading to a growing proportion of the population being aged 65 and above. The aging process is associated with organ function deterioration, increased risks of age‐related diseases, and a decrease in the quality of life for older adults. While there is ongoing debate about whether aging should be considered a disease or a natural physiological process, understanding the reasons for aging, identifying and measuring aging, and intervening in the aging process have become key topics in current medical research. Recent studies indicate that the rate of organ aging varies among individuals, with blood vessels being one of the organs that age first. Vascular aging increases the risk of cardiovascular diseases, overall mortality, and shortens lifespan. Therefore, in‐depth research on vascular aging is crucial for understanding its impact on vascular and multi‐organ function. In this review, we discuss the phenotypes caused by vascular aging, mechanisms of aging in various vascular cells, and potential therapeutic strategies for vascular aging.

## Graphical abstract

The phenotypes of vascular aging vary across different blood vessels. Endothelial dysfunction is observable in the endothelium of all vessels, while vascular structural remodeling primarily occurs in arteries. A decline in organ vascular density mainly affects arterioles, venules, and capillaries, whereas thrombosis predominantly occurs in the capillaries and venous system.

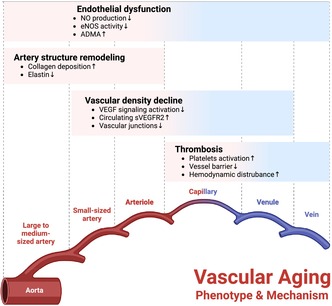

## 1 Introduction

Aging is an irreversible biological process that leads to the deterioration of multiple organ functions. The aging process manifests itself in various ways, including cellular damage, changes in gene expression, and the accumulation of metabolic waste products [1]. These factors contribute to the decline in organ function and ultimately affect overall health. Even though research on aging has spanned over a century, uncovering various conserved mechanisms of aging and extensively studying it in model organisms like yeast, nematodes, and mice, the comprehension of vascular aging still falls significantly short compared to systemic aging.

Blood vessels are a highly heterogeneous organ, comprised of arteries, microvascular, veins, and specialized vessels such as hepatic sinusoids, blood–brain barrier vessels, pulmonary alveolar capillary barrier, and gas exchange capillaries. Structurally, large arteries and veins exhibit a three‐layer composition: the outer layer, middle layer, and inner layer. In contrast, microvascular typically consist of a uniform single layer of endothelial cells adhered to the basement membrane, facilitating efficient tissue oxygenation and material exchange between blood and tissues. The complexity of the vascular system lies not only in its physical structure but also in its intricate functional roles within the body's circulatory and homeostatic processes. Arterial blood, enriched with oxygen and essential nutrients, serves as the lifeline for various tissues and organs, ensuring their metabolic needs are met. Microvascular, with their thin walls and proximity to tissues, enable the vital exchange of gases, nutrients, and waste products, thus supporting cellular functions and maintaining tissue health. Veins, on the other hand, play a crucial role in removing metabolic by‐products and transporting deoxygenated blood back to the heart for reoxygenation. This intricate interplay between arteries, microvascular, and veins illustrates the dynamic nature of the vascular system and its fundamental contribution to overall physiological balance.

While studies have made remarkable strides in elucidating the fundamental processes that govern aging at the cellular and organismal levels, the intricate mechanisms underlying vascular aging present a unique challenge due to the multifaceted nature of the cardiovascular system and its susceptibility to both intrinsic and extrinsic factors. Understanding vascular aging is crucial as it underpins the development and progression of many age‐related diseases, such as cardiovascular disorders and neurodegenerative conditions [2]. By delving deeper into the intricacies of vascular aging, researchers not only enhance our knowledge of aging as a whole but also provide crucial insights into potential therapeutic interventions that can target age‐related vascular dysfunctions and ultimately improve healthspan and quality of life. Despite the vast body of knowledge accumulated in the field of aging research, it remains imperative to bridge the gap in our understanding of vascular aging to fully grasp the complexities of the aging process and its implications on overall health and longevity.

## 2 Phenotypes and Mechanisms of Vascular Aging

### 2.1 Endothelial Dysfunction

Endothelial cells are a crucial component of the cardiovascular system, including arteries, veins, capillaries, lymphatic vessels, and endocardium, acting as a barrier between blood and the surrounding tissues while also actively participating in processes that regulate cardiovascular function [3]. These specialized cells come into direct contact with blood flow, allowing them to sense changes in shear stress and release nitric oxide (NO) to control vasodilation [3]. The interaction between endothelial cells and blood flow plays a pivotal role in maintaining cardiovascular homeostasis. When blood flows over the endothelial cells, shear stress is generated, causing the cells to respond by releasing NO, a potent vasodilator. NO acts on smooth muscle cells in the vessel walls, promoting relaxation and vasodilation, ultimately leading to increased blood flow to meet the metabolic demands of the tissues [4].

Aging disrupts the nitric oxide synthesis mechanism, leading to impaired flow‐dependent vasodilation and resulting in vascular stiffness and elevated blood pressure. As individuals age, there is a notable decline in the production and bioavailability of NO due to multiple intricate mechanisms. One key aspect contributing to this decline is the reduced activity of endothelial nitric oxide synthase (eNOS) enzyme, which is responsible for NO synthesis in the endothelial cells of blood vessels [5, 6, 7]. The decreased eNOS activity is often associated with age‐related vascular dysfunction and cardiovascular diseases. The increased cleavage of endothelial BACE1 (β‐site amyloid precursor protein cleaving enzyme 1) elevates the tight junction protein occludin, leading to the accumulation and internalization of caveolin‐1 on the membrane. Subsequently, intracellular caveolin‐1 binds with eNOS, causing a 23% reduction in the phosphorylation level of eNOS at S1177 in response to β‐amyloid peptides. This results in endothelial dysfunction and increases the risk of hypertension and Alzheimer's disease [8].

Furthermore, oxidative stress, a common feature of aging, can lead to the uncoupling of eNOS, shifting its function from NO production to superoxide anion generation [9, 10]. This phenomenon not only diminishes NO availability but also promotes oxidative damage and inflammation, exacerbating age‐related pathologies. In addition to eNOS dysfunction, another mechanism that impedes NO synthesis in aging is the upregulation of asymmetric dimethylarginine (ADMA), an endogenous inhibitor of eNOS [11, 12]. Elevated levels of ADMA competitively inhibit eNOS activity, reducing the production of NO and contributing to endothelial dysfunction [13]. The dysregulation of ADMA metabolism in the aging process underscores the intricate interplay between endogenous NO inhibitors and eNOS function.

Moreover, age‐related changes in the expression and function of key regulatory proteins involved in NO synthesis pathways also contribute to impaired NO bioavailability. For instance, age‐related alterations in the expression of heat shock protein 90 (Hsp90), an essential chaperone protein for eNOS, can disrupt the proper folding and function of eNOS, leading to decreased NO production [14, 15, 16]. The decline in Hsp90 levels with age may compromise the stability and activity of eNOS, further exacerbating NO synthesis defects in aging individuals [17]. Additionally, age‐associated alterations in the activity of caveolin‐1, a negative regulator of eNOS, can impact eNOS localization and function within the endothelial cells, affecting NO synthesis capacity [18, 19]. The dysregulation of caveolin‐1‐mediated eNOS inhibition serves as another critical mechanism underlying the age‐related decline in NO bioavailability [20, 21]. Furthermore, the age‐related increase in inflammatory mediators and cytokines can disrupt NO synthesis mechanisms through multiple pathways. Chronic low‐grade inflammation, commonly observed in aging individuals, can induce the expression of inducible nitric oxide synthase (iNOS), an enzyme responsible for excessive NO production under inflammatory conditions [22]. The upregulation of iNOS in response to heightened inflammatory signaling not only leads to an imbalance in NO levels but also contributes to oxidative stress and tissue damage in aged individuals [23]. The interaction between inflammatory cytokines and eNOS activity can further exacerbate NO synthesis impairment in aging, highlighting the intricate crosstalk between inflammation and NO signaling pathways.

Cellular senescence is a significant contributor to endothelial dysfunction, affecting not only vascular cells but also surrounding cells and immune cells. Senescent endothelial cells exhibit decreased proliferation and migration, reduced NO signaling, increased production of inflammatory cytokines, and a diminished response to inflammatory stimuli [24, 25]. Recent research indicates that senescent macrophages in aged murine hindlimb muscle promote endothelial dysfunction by increasing the production of the anti‐angiogenic factor VEGFA‐165B (vascular endothelial growth factor), thereby impairing ischemia‐induced angiogenesis in aged murine hindlimbs [26]. Conversely, senescent endothelial cells can impair angiogenesis by suppressing VEGFA secretion from macrophages via miR‐146 transfer [27]. These findings highlight the intricate crosstalk between vascular aging and endothelial dysfunction.

### 2.2 Arterial Structure Remodeling

**FIGURE 1:**
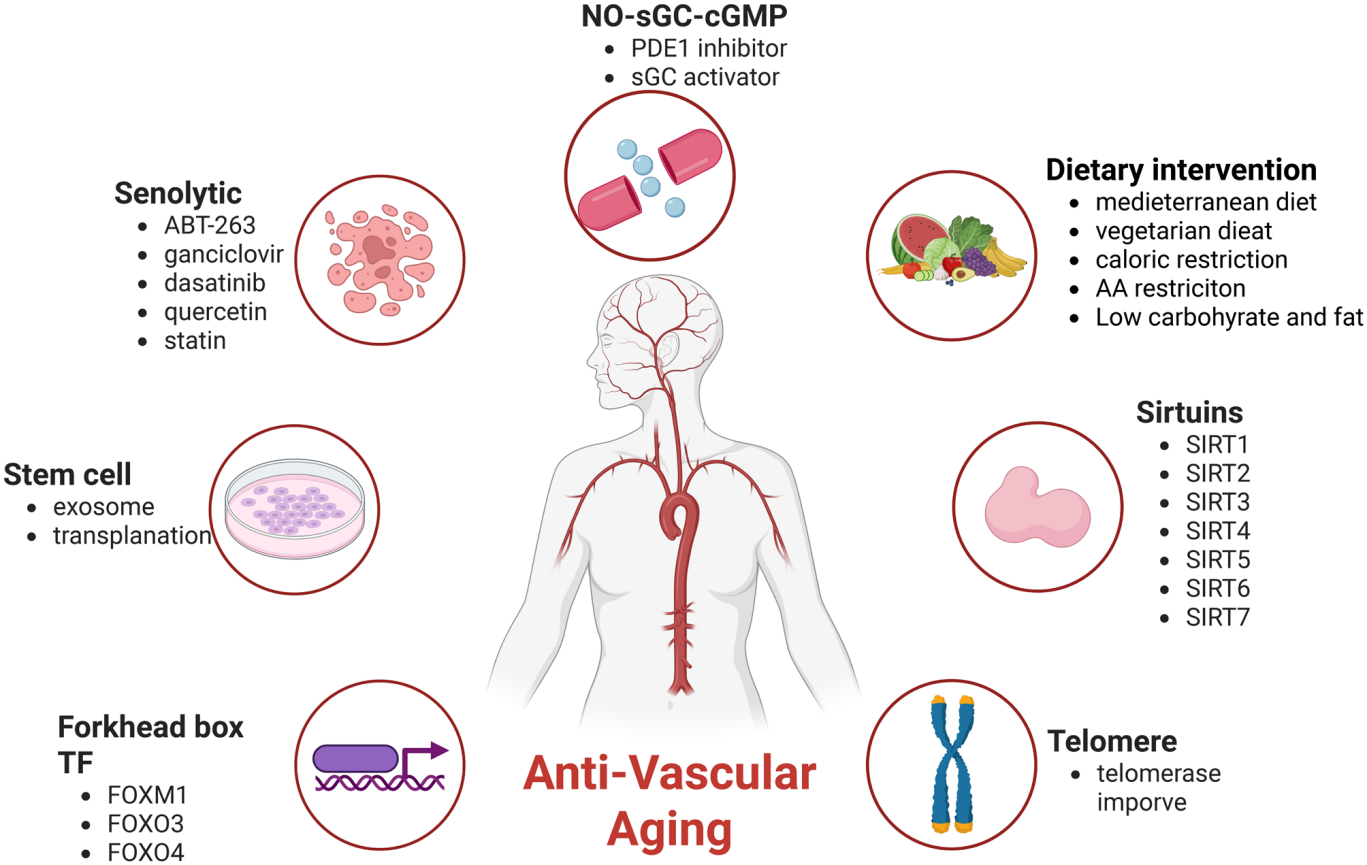
Potential therapeutic targets and strategies for vascular aging. This figure illustrates seven potential research directions for vascular aging treatments, each of which is discussed in detail in this review.

The arterial wall is composed of the intima (consisting of endothelial cells and elastic laminae) and the media (comprising smooth muscle, elastic tissue, and connective tissue). Data from clinical and preclinical studies conducted in the last century indicate that as individuals age beyond maturity and progress into aging, there is a notable phenomenon of progressive dilatation and elongation of the aorta [28]. In a large population‐based study conducted in the United States, it was found that the average diameter of the aorta in 80‐year‐olds increased by 15%–23% compared to 50‐year‐olds [29]. The reduced distensibility of the arteries leads to changes in their cushioning function, resulting in a diminished ability to absorb the pressure generated during cardiac systole and recoil effectively during diastole [30, 31]. The age‐related decline in the distensibility of the aorta and large arterial vessels is predominantly due to intrinsic changes, such as the thickening of the media and intima layers of the vessel walls (Figure 1). Individuals aged 75 and over show an average increase of approximately 19% in intima thickness and over 96% in media thickness in the carotid artery compared to those aged under 45 [32]. This thickening ultimately leads to a reduction in vascular compliance and an increase in stiffness.

The thickening of artery walls due to aging is a complex process involving multiple factors. One of the primary reasons for this phenomenon is the enhanced deposition of collagen within the arterial walls as individuals age. Collagen, a structural protein crucial for maintaining the integrity and strength of tissues, is naturally prevalent in the walls of blood vessels. As blood vessels age, there is an upregulation of collagen production, particularly types I and III collagen, leading to increased deposition in the extracellular matrix [33, 34, 35]. The causes of abnormal collagen metabolism due to aging are complex. TGFβ (transforming growth factor‐β) is a key molecule in arterial remodeling and has been found to promote adventitial collagen I and III in aged mice [36]. Disturbed flow can upregulate the expression of TSP1 (thrombospondin‐1) in the human aorta, guiding collagen type I/IV synthesis and deposition through the TGFβ1 signal promoting arterial stiffening and vascular aging [37]. The absence of Wnk1 (with‐no‐lysine K kinase 1) in VSMCs (vascular smooth muscle cells) resulted in increased collagen content, TGFβ activation, and artery remodeling [38]. TGFβ induces IL‐11, which triggers ERK‐dependent secretion of collagens and MMPs (matrix metalloproteinases) to promote cardiovascular remodeling [39]. Silencing the fatty acid transporter protein CD36 inhibits the expression of TGFβ protein and the synthesis of type I collagen, while promoting the expression of contractile proteins, maintaining VSMCs in a contracted state, and preventing arterial remodeling. This suggests that fatty acid metabolism may be involved in collagen synthesis [40].

The reduced degradation and accumulation of collagen are not only consequences but also causes of vascular cell senescence. Mice expressing the anti‐collagenase form of type I collagen exhibit progressive dorsal kyphosis, age‐related hypertension, and reduced lifespan due to excessive collagen deposition, which leads to increased expression of senescence markers p16, p21, and β‐galactosidase staining in vascular smooth muscle cells and fibroblasts [41].

Furthermore, arterial stiffness rise leads to vascular mechanical stress microenvironment remodeling, accelerating the senescence process. The increased stiffness of blood vessels promotes the nuclear entry of mechanosensitive transcription factor SOX9 (sex determining region Y‐box 9), leading to enhanced collagen expression in vascular smooth muscle cells and reduced contractility, resulting in extracellular matrix remodeling and accelerating vascular cell senescence within the matrix [42]. In pulmonary arterial adventitial fibroblasts, inflammation, hypoxia, and mechanical stress converge to activate the transcriptional coactivators YAP (yes‐associated protein) and TAZ (transcriptional co‐activator with PDZ‐binding motif). This activation leads to YAP and TAZ driving glutamine and serine catabolism, which supports proline and glycine anabolism, subsequently enhancing collagen biosynthesis [43]. Mechanosensitive channel PIEZO1 upregulates microRNA‐582‐5p in vascular smooth muscle cells, leading to collagen deposition through the PTEN/PI3K/Akt signaling pathway, ultimately causing arterial stiffness, thickening, fibrosis, and elevated senescence markers [44].

Brain endothelial glycocalyx dysregulation has been identified as a pathological hallmark of blood–brain barrier (BBB) dysfunction in aging and neurodegenerative diseases. A recent study reveals a conserved impairment of mucin‐type O‐glycosylation in brain endothelia under these pathological conditions [45]. Experimental evidence demonstrates that genetic attenuation of this post‐translational modification increases BBB permeability and elevates susceptibility to cerebral hemorrhage [45]. Notably, AAV‐mediated (adeno‐associated virus) restoration of endothelial O‐glycan biosynthetic enzymes was shown to rescue BBB integrity, reduce neuroinflammatory markers, and improve cognitive performance in aged murine models [45]. This suggests that understanding vascular remodeling requires considering factors beyond the intima and adventitia. Intimal attachments and structures external to the adventitia may also significantly influence vascular aging.

### 2.3 Vascular Density Decline

Aging results in the loss of capillaries, arteries, and peripheral cells in various organs such as the kidneys, muscles, spleen, bone, and thymus in aging humans and mice [46, 47, 48]. This vascular loss occurs earlier than organ aging signal emergence and acts as a driving force for the aging process [46, 49, 50]. The main reason for organ vascular bed degeneration due to aging is weakened VEGF signal. Mice with specific VEGFR2 deficiency exhibit reduced capillary network formation and pericyte coverage, promoting pericyte differentiation into fibroblasts that exacerbate age‐dependent organ and vascular fibrosis [46]. Interestingly, in aging mice, the levels of VEGFA in circulation not only did not decrease but increased by approximately 1.5‐fold. However, this increase did not improve the weakened VEGF signal, as evidenced by decreased VEGFR2 phosphorylation and organ microvascular degradation. This may be due to the competitive inhibition of VEGFA by elevated sVEGFR2 in circulation [51].

The restructuring of the extracellular matrix and collagen is a determining factor for vascular density and function. Increased hardness activates β‐Catenin through ROCK/pMLC/FAK, disrupts vascular junctions to increase permeability, and leads to vascular rarefaction [47]. The quantity and potential for specialization of type II collagen‐positive progenitor cells in the long bone and knee joints decreased as individuals aged, resulting in skeletal blood vessels' decline [52].

It is worth noting that while the decrease in microvascular density mostly happens in the later stages of aging, resulting organ dysfunction may occur before vascular degeneration appears. Degeneration of cardiac microvessels was observed in 22‐month‐old mice, while cardiac nerve degeneration due to aging microvessels occurred as early as 16 months of age. Aging microvessels in the heart downregulate miR‐145, leading to increased secretion of SEMA3A (semaphorin‐3A) by the cardiac microvessel endothelium, causing axonal damage in the cardiac nerves and culminating in changes associated with cardiac neural aging [53]. In addition, the decreased coverage of pericytes in the microvessels of aged and dysfunctional hearts is associated with reduced longevity gene BPIFB4 (bacterial permeability‐increasing protein family B member 4). Maintaining the expression of BPIFB4 can enhance microvessel density and pericyte coverage in aging hearts, thereby improving cardiac function [54, 55]. However, the decrease in microvessel coverage is not universally observed, as in aging pancreas, testes, and thyroid, only reductions in microvessels and arteries are seen, with no decrease in perivascular cells [56].

### 2.4 Thrombosis

The elderly are at greater risk of developing thromboembolism, which leads to serious health problems and death [57]. The rate of thrombus formation in the carotid artery and inferior vena cava of aged mice is faster than that in young mice, indicating higher coagulation activity [58, 59]. Aging causes activation of platelets and the coagulation system, which is the main mechanism leading to the formation of blood clots. The hyperactive mTORC1 (mammalian target of rapamycin complex 1) was found in platelets and megakaryocytes (MKs) of aged mice, resulting in increased mean platelet volume (MPV) and platelet activation. Treatment with rapamycin, an mTORC1 inhibitor, significantly reduced the likelihood of experimental deep vein thrombosis (DVT) in aged mice [60]. It is noteworthy that aging causes stem cells to differentiate into a specific population of megakaryocyte progenitor cells that parallel the typical platelet differentiation pathway. This cell population exhibits enhanced proliferation and platelet production abilities. The coexistence of two platelet pools in aging mice leads to an increase in platelet count. The functionally enhanced platelets induced by aging have a higher thrombus formation capability, significantly increasing in vivo thrombus formation [61].

Endothelial cells act as a physical barrier that lines the interior of blood vessels, regulating the exchange of molecules between the blood and surrounding tissues. This barrier function is essential in preventing the activation of the coagulation cascade and maintaining blood fluidity. One significant aspect of aging is its effect on the weakening of the blood vessel barrier, which can lead to an increased risk of thrombus formation. The other age‐related changes in the vasculature, including reduced vascular elasticity and increased inflammation, create an environment conducive to thrombus formation [62]. With advancing age, the endothelial cells exhibit decreased production of NO and increased expression of adhesion molecules, fostering a proinflammatory and prothrombotic environment [63, 64]. These alterations in endothelial function not only compromise the integrity of the blood vessel barrier but also promote the adhesion of circulating platelets and inflammatory cells, further fueling the process of thrombus formation [65].

Furthermore, aging‐related changes in the composition and structure of the vessel wall can contribute to the pathogenesis of thrombosis. Collagen and elastin fibers within the vessel walls undergo modifications with age, leading to increased stiffness and reduced elasticity [66, 67]. These alterations can disrupt blood flow dynamics and promote the formation of turbulent blood flow patterns that facilitate platelet aggregation and thrombus formation [68]. The deposition of atherosclerotic plaques in the vessel walls, a common feature of aging, can also serve as sites for thrombus initiation, particularly in the presence of endothelial damage and prothrombotic conditions.

## 3 Potential Therapeutic Targets and Strategies

### 3.1 Dietary Intervention

Research suggests that adopting a healthy diet can help to mitigate the effects of vascular aging and promote cardiovascular health. In particular, consuming a diet rich in fruits, vegetables, whole grains, and lean proteins has been shown to have a positive impact on vascular health. These foods are high in antioxidants, vitamins, and minerals, which can help to reduce inflammation, oxidative stress, and arterial stiffness—all of which are key factors in vascular aging. Due to its low economic cost and high feasibility, dietary intervention has become the most promising strategy for combating vascular aging. A recent 30‐year study of over 100,000 individuals confirms the link between diet and healthy aging [69, 70]. However, there is diversity in the effects and mechanisms of different dietary patterns on vascular aging. It is important to note that due to differences in genomic and metabolic patterns between humans and animals such as mice, worms, and monkeys, dietary interventions and metabolic‐targeting therapies like those involving mTOR, which show strong anti‐aging effects in animals, have not achieved the expected results in humans. Therefore, our focus is not on these animal studies but rather on dietary interventions that may potentially impact human vascular aging phenotypes. Although these interventions may help improve vascular aging, it is regrettable that they do not appear to extend lifespan. The irreversibility of vascular aging may contribute to this outcome, as dietary control is typically implemented in clinical settings for individuals with high‐risk factors for diseases. In these individuals, vascular aging may have already been activated and accelerated. While dietary control can be beneficial for metabolic diseases, it does not aid in reversing vascular aging.

#### 3.1.1 Mediterranean Diet

The Mediterranean diet is a traditional dietary pattern in the Mediterranean region known for its key components: high consumption of seasonal fruits and vegetables, nuts, cereals (primarily whole grain), legumes, olive oil, and fish. Additionally, the diet includes moderate alcohol and dairy product intake while minimizing meat and meat product consumption [71, 72]. The randomized controlled trials (RCTs) confirmed the beneficial effects of the Mediterranean diet on human vascular aging, including reducing PWV (pulse wave velocity) and carotid intima‐media thickness (cIMT), as well as improving FMD (flow‐mediated dilation) [73, 74, 75, 76, 77, 78, 79].

#### 3.1.2 Vegetarian Diet

Vegetarian diets involve reducing or eliminating the consumption of animal products. They typically consist of grains, legumes, fruits, vegetables, and nuts, while being low in added sugars, salt, cholesterol, and saturated fat. Cross‐sectional studies show that a vegetarian diet does not improve cIMT, FMD, or PWV [80, 81, 82]. However, prospective studies have found that a vegetarian diet can reduce cIMT in Asian populations [83]. The effects of a vegetarian diet on vascular aging are controversial and require further research for a definitive answer.

#### 3.1.3 Caloric Restriction (CR)

Caloric restriction refers to a dietary pattern in which an individual's daily energy intake is reduced relative to normal expenditure, while ensuring adequate nutrition without malnutrition. Intermittent fasting, such as the 16:8 or 5:2 diet and alternate‐day fasting, are optional approach to reducing overall calorie intake [84, 85]. While CR undeniably plays a powerful role in extending lifespan in model animal studies, its effectiveness in slowing vascular aging in clinical research has fallen short of expectations. Research has shown that CR appears to have a greater impact on vascular aging in obese patients, increasing FMD and reducing PWV [86, 87, 88, 89, 90]. However, for healthy individuals, while CR may not improve FMD, it can reduce blood pressure and PWV, as well as lower systemic inflammation [91, 92, 93]. Therefore, the vascular protective effects of CR are more attributed to the benefits of weight loss in obese patients rather than CR itself. The benefits of weight loss through exercise are comparable to those of CR in terms of vascular protection [94].

#### 3.1.4 Low Carbohydrate and Fat Diet

High‐carbohydrate and high‐fat diets are significant risk factors for metabolic syndrome and obesity, driving vascular aging. Contrary to expectations, low‐carb or low‐fat diets do not improve vascular aging in patients with obesity or metabolic syndrome, showing minimal impact on cIMT, PWV, and FMD [95, 96, 97].

#### 3.1.5 Protein and Amino Acid Restriction Diet

Proteins and amino acids play crucial roles in various physiological processes within the human body, including maintaining vascular health and function. Excessive intake of branched‐chain amino acids or isoleucine can lead to increased food consumption, obesity, and shortened lifespan [98, 99]. Clinical experiments have shown that restricting methionine and cysteine intake reduces human body weight and cholesterol levels while increasing circulating FGF21 (fibroblast growth factor), a regulatory molecule highly associated with improving aging [100]. In pre‐clinical studies, sulfur amino acid restriction acts as a proangiogenic cue, enhancing VEGF expression, migration, and sprouting in vitro endothelial cells and boosting capillary density in mouse skeletal muscle in vivo through the GCN2/ATF4 amino acid deprivation response pathway, without involving hypoxia or HIF1α (hypoxia inducible factor) [101]. Restricting the intake of dietary glutamic acid and serine or using drugs to block their biosynthesis can reduce vascular collagen content and improve arterial stiffness [43]. Leucine supplementation improved aging‐induced vascular dysfunction and remodeling in middle‐aged mice [102].

### 3.2 Sirtuins

SIRTs, a class of proteins known for their regulatory functions in various physiological processes, have garnered increasing attention in the realm of aging research due to their significant impact on lifespan and overall healthspan. The SIRTs constitute 7 proteins with nicotinamide adenine dinucleotide‐dependent deacetylase or adenosine diphosphate‐ribosyltransferase activity. SIRT1 and SIRT2 are found in the nucleus and cytoplasm, while SIRT3, SIRT4, and SIRT5 are mitochondrial. SIRT6 and SIRT7 are localized in the nucleus [103]. Due to their differing distributions within cells, their substrate selectivity also varies. Although SIRT proteins share functional similarities, each SIRT is indispensable, as the absence of any one SIRT leads to cellular aging [104]. This suggests that SIRTs collectively determine the cellular aging homeostasis, much like the staves of a barrel.

#### 3.2.1 SIRT1

This protein functions as an NAD+ ‐dependent deacetylase, which means it plays a crucial role in cellular metabolism and stress response by removing acetyl groups from various substrates, including histones and non‐histone proteins. SIRT1 expression decreases progressively with age in the endothelial cells and vascular smooth muscle cells of the aorta [102, 105]. The knockout of endothelial SIRT1 mimicked age‐related capillary degeneration, while overexpression of SIRT1 rescued organ capillary neovascularization, indicating that the decrease in SIRT1 is not only a consequence of vascular aging but also a driving force for accelerating vascular aging [106]. Furthermore, providing the NAD+ precursor NMN improved the capillary density in aging mice. It suppressed NOTCH activation mediated by SIRT1, and knocking out SIRT1 eliminated the effects of NMN [106]. SIRT1 was up‐regulated in VSMCs in response to CR and removing SIRT1 specifically in smooth muscle cells eliminated CR's preventive effect on abdominal aortic aneurysm formation [107]. CR also increases the production of NO by normalizing ROS, improving cardiovascular damage caused by metabolic abnormalities. DNA damage leads to the downregulation of LARP7 (La‐related protein), reducing SIRT1 deacetylase activity and increasing acetylation of p53 and NF‐κB (p65), which boosts their transcriptional activity and speeds up aorta senescence and atherosclerosis [108]. Spermidine can inhibit endoplasmic reticulum stress in vascular smooth muscle cells by upregulating SIRT1, thereby reducing VSMC osteogenic differentiation and vascular calcification [109]. The binding of SIRT1 to the promoters of p66Shc and arginase 2 decreases with aging, indicating that aging reduces SIRT1's inhibitory effect on oxidative stress, leading to dysfunction in visceral arterioles and administration of the SIRT1 activator SRT1720 can improve arterial aging [110].

#### 3.2.2 SIRT2

SIRT2 exerts its effects through various mechanisms, including the regulation of oxidative stress, inflammation, and cellular senescence, all of which are pivotal in the aging heart and vascular system. Although SIRT2 is the most abundantly transcribed sirtuin in the human aorta, research on its role in cardiovascular aging is limited. SIRT2 activity decreased in aged aortas, and the loss of SIRT2 accelerated vascular aging. In older mice, SIRT2 deficiency worsened age‐related arterial stiffness and constriction‐relaxation dysfunction, along with aortic remodeling [34]. Unlike SIRT1, which regulates p66Shc expression through epigenetic mechanisms, SIRT2 inhibits p66Shc activation and mtROS production by deacetylating p66Shc at lysine 81, thereby accelerating vascular aging [34]. SIRT2 knockout significantly worsened cardiac hypertrophy and fibrosis while reducing ejection fraction and fractional shortening in 24‐month‐old mice [111]. SIRT2 activates AMPK by deacetylating the kinase LKB1, and the loss of SIRT2 diminishes AMPK activation and contributes to aging‐related cardiac hypertrophy [111]. SIRT2 was also identified as a key molecular factor that decreases in aged monkey hearts. Inducing SIRT2 deficiency in human pluripotent stem cell‐derived cardiomyocytes replicated key senescence features of primate heart aging [112]. Depletion of SIRT2 in human cardiomyocytes caused STAT3 hyperacetylation, which activated CDKN2B (p15) transcription and subsequently triggered cardiomyocyte degeneration [112].

#### 3.2.3 SIRT3

As a mitochondrial deacetylase, SIRT3 is instrumental in modulating various metabolic pathways that are crucial for maintaining cellular homeostasis and energy metabolism within cardiovascular tissues. Global SIRT3 depletion in mice leads to vascular hypertrophy and increases vascular permeability and inflammation, and reduces telomerase expression, accelerating vascular senescence and age‐dependent hypertension [113]. The deletion of SIRT3 specifically in cardiomyocytes also leads to age‐dependent hyperacetylation and cardiac hypertrophy in 10‐month‐old mice [114]. Aging reduces CX43 in the blood–brain barrier, which increases nuclear localization of PARP1 (poly (ADP‐ribose) polymerase). This leads to decreased NAD+ levels, triggering autophagy abnormalities and SIRT3 degradation, ultimately compromising the structural integrity of the blood–brain barrier [115]. Furthermore, the interaction between mitochondrial SIRT1 and SIRT3 is enhanced, providing adaptive protection against further mitochondrial dysfunction in aging hearts by deacetylating long‐chain acyl‐CoA dehydrogenase (LCAD) during ischemic injury [116]. The recent study showed that SIRT3 prevents endothelial cell aging by activating DHRS2‐PKCδ, which inhibits the translocation of uncoupled eNOS to mitochondria [117].

#### 3.2.4 SIRT4

Although SIRT4 is highly expressed in the heart and aorta, research on SIRT4 in cardiovascular aging is limited, which hinders the understanding of its role in this process. In aged mice, SIRT4 mRNA expression remains unchanged in the aorta but decreases in the aortic valve [118]. Surprisingly, SIRT4 promotes Ang II‐induced cardiac hypertrophy and remodeling via enhanced oxidative stress and inhibits SIRT3‐mediated manganese superoxide dismutase deacetylation [119]. It is noteworthy that neither the knockout nor the overexpression of SIRT4 alters baseline blood pressure or prevents Ang II‐induced hypertension, suggesting that SIRT4 may have no impact on vascular aging and remodeling [119]. In vitro, upregulation of SIRT4 in HUVECs alleviated sepsis‐related inflammatory pathways, reduced mitochondrial stress, and prevented apoptosis, pyroptosis, and autophagy [120, 121]. This indicates that SIRT4 may provide stronger protection against acute severe vasculitis than against chronic inflammation induced by aging.

#### 3.2.5 SIRT5

SIRT5 is primarily localized in the mitochondria, where it plays a pivotal role in regulating metabolic processes and maintaining mitochondrial integrity. SIRT5 transgenic mice exhibited faster arterial thrombus formation after endothelial‐specific damage, whereas SIRT5KO mice showed reduced arterial thrombosis [122]. Increased SIRT5 expression enhances mitochondrial function and boosts angiogenic capacity in endothelial progenitor cells [123]. SIRT5KO mice develop hypertrophic cardiomyopathy, indicated by increased heart weight relative to body weight and reduced shortening and ejection fractions [124]. This indicates that SIRT5 plays a crucial role in maintaining myocardial metabolic homeostasis, but its direct link to cardiac aging has yet to be confirmed. Although SIRT5 has a limited role in cardiovascular aging, SNP analysis suggests a link between SIRT5 and longevity, indicating that SIRT5 may extend lifespan by regulating aging in other tissues and organs [125].

#### 3.2.6 SIRT6

SIRT6, a member of the NAD +‐dependent sirtuin family, has garnered significant attention for its crucial role in extending lifespan and improving age‐related cardiovascular functions [126, 127]. Clinical studies have shown that SIRT6 expression is related to stroke outcomes, as SIRT6 helps maintain the integrity of the blood–brain barrier. Mice lacking SIRT6 exhibit larger infarct areas during stroke, confirming SIRT6's crucial role in vascular homeostasis [128]. SIRT6 was markedly downregulated in the radial artery tissue of patients with vascular calcification, and SIRT6‐transgenic (SIRT6‐Tg) mice showed alleviated vascular calcification [129]. Mechanistically, SIRT6 directly binds to and deacetylates RUNX2, activating XPO1 nuclear export and promoting RUNX2 degradation via the ubiquitin‐proteasome system, thereby inhibiting osteogenic differentiation of smooth muscle cells [129]. SIRT6 also inhibited GATA6 transcription by deacetylation and increased the degradation of the transcription factor Nkx2.5, thereby rescuing senescent and calcified aortas [130]. In cardiomyocytes, SIRT6 binds with PPARα to upregulate fatty acid oxidation molecules such as CD36 and CPT1, thereby improving fatty acid metabolism homeostasis and helping cardiomyocytes resist aging stress [131].

#### 3.2.7 SIRT7

SIRT7, the latest identified member of the SIRTs, maintains physiological homeostasis and protects against aging by safeguarding genomic integrity and dynamically sensing and modulating stress [132]. The SIRT7 knockout mice exhibited an extended average and maximum lifespan, delayed age‐related mortality, and improved glucose tolerance [133]. Targeting the vascular endothelium with SIRT7 gene therapy enhances neovascularization, reduces aging characteristics, and extends the lifespan in HGPS (Hutchinson‐Gilford progeria syndrome) mice [134]. Targeting SIRT7 specifically in the endothelium also alleviates pulmonary hypertension by deacetylating KLF4 K228 [135].

Although substantial evidence indicates that SIRTs are key molecules in delaying cardiovascular aging, therapeutic strategies aimed at enhancing SIRT activity still require rigorous evaluation, as SIRTs can exacerbate disease progression in certain pathological conditions. In the hypoxic microenvironment of arterial plaques, SIRT6 can promote angiogenesis by activating HIF1α. However, the high oxidative stress within the plaques causes SIRT6 to acetylate H3K56, inhibiting catalase and exacerbating oxidative stress, which damages the newly formed blood vessels. This dual effect leads to the excessive growth of structurally and functionally abnormal vessels within the plaques, resulting in plaque hemorrhage and rupture [136].

### 3.3 Telomere

As individuals age, the efficiency of DNA repair mechanisms declines, leading to an accumulation of DNA damage. This accumulation initiates a cascade of molecular events, notably the activation of pro‐inflammatory pathways and oxidative stress. These processes, in turn, exacerbate vascular dysfunction, highlighting the interconnected nature of cellular damage and systemic health deterioration in the aging population.

Telomeres, which are repetitive nucleotide sequences located at the ends of eukaryotic chromosomes, are essential for maintaining genomic stability. They achieve this by protecting DNA from degradation and preventing the end‐to‐end fusion of chromosomes. Additionally, telomeres address the end‐replication problem, a challenge where DNA polymerases cannot fully replicate the 3′ ends of linear DNA molecules, leading to progressive telomere shortening with each cell division [137]. This shortening is counteracted by the enzyme telomerase, a ribonucleoprotein reverse transcriptase that extends telomeres by adding telomeric repeats to the 3′ end of chromosomes, thereby promoting cellular longevity [138]. However, in most somatic cells, telomerase activity is either insufficient or absent, resulting in gradual telomere shortening that ultimately triggers replicative senescence or apoptosis [139, 140]. Telomeres are particularly vulnerable to DNA damage, which triggers a DNA damage response that subsequently induces the expression of inflammatory genes [141, 142]. This response is closely associated with the development of SASP (senescence‐associated secretory phenotype) [143]. The paracrine factors released by SASP have been demonstrated to induce senescence in neighboring cells, thereby contributing to the progression of age‐related diseases [144].

Telomeres have a significant impact on vascular cells, including endothelial and smooth muscle cells, especially in premature vascular aging disease [145]. Truncation mutations in lamin A are responsible for HGPS, a severe form of early‐onset premature aging [146]. In both mice and humans, HGPS results in shortened lifespans. In humans, the primary causes of death are stroke and ischemic heart disease, both resulting from premature vascular aging [147, 148, 149]. Cells from HGPS patients exhibit accumulated DNA damage, chromosomal instability, increased sensitivity to DNA‐damaging agents, and accelerated telomere attrition [150]. Transducing telomerase mRNA into endothelial cells extended telomere length, enhanced replicative capacity, restored nitric oxide generation and angiogenesis, and increased the lifespan of HGPS mice by 20% [151].

Furthermore, the interaction between telomeres and cellular aging extends beyond DNA damage and repair. It is also closely related to mitochondrial dysfunction, protein homeostasis imbalance, and nutrient sensing, as thoroughly discussed in previous reviews [138]. It is important to note that the telomere length in mice is approximately five times that of humans. Consequently, many scientists believe that telomere attrition plays a negligible role in vascular aging in mice. This belief may lead to an underestimation of the potential clinical significance of telomere‐targeted therapies when tested in mice. In summary, these studies provide evidence and potential delivery methods for using telomere extension to improve vascular aging.

### 3.4 Senolytic Therapy

Senolytics, compounds that target and eliminate senescent cells, enhance specific aspects of physiological function as individuals age [152]. Ganciclovir and ABT‐263 reduced aortic stiffness, collagen deposition, and SASP in aging mice, leading to improved nitric oxide biosynthesis and vascular compliance [153]. Dasatinib and quercetin treatment in old mice led to a reduction in senescent cells, which in turn resulted in a rescue of total nerve density and restoration of both sympathetic and sensory nerves in the hearts. Furthermore, by inhibiting age‐induced increases in capillary perimeter and enhancing the outgrowth of aortic endothelial cells ex vivo, they also effectively abrogated the age‐associated impairment of endothelial function [53]. Statins, a commonly used cardiovascular lipid‐lowering drug, have been found to selectively eliminate senescent cells without harming non‐senescent cells [154].

### 3.5 Forkhead Box Transcription Factor

The forkhead transcription factors play a pivotal role in the regulation of aging and longevity, acting as critical mediators of cellular homeostasis and stress resistance.

#### 3.5.1 FOXM1

FOXM1 is an important mediator of endothelial regeneration in young mice, and transgenic FOXM1 expression or in vivo endothelium‐targeted nanoparticle delivery of the FOXM1 gene reactivated endothelial regeneration, normalized vascular repair, resolved inflammation, and promoted survival in aged mice [155]. The restoration of FOXM1 also alleviates endothelial cell senescence caused by SIRT6 deficiency [156]. In vivo, globe knock‐in FOXM1 mitigates several cellular aging hallmarks and molecular and histopathological progeroid features in the short‐lived Hutchison‐Gilford progeria mouse model, significantly extending its lifespan [157, 158].

#### 3.5.2 FOXO3 (FOXO3A)

Clinical studies have observed that individuals with the FOXO3 rs2802292 longevity genotype have a lower risk of hypertension and dementia in old age, suggesting a protective role of FOXO3 against vascular aging [159]. The overexpression of FOXO3 enhances vascular cells' resistance to aging and oxidative stress by downregulating CSRP1, thereby promoting post‐ischemic vascular regeneration in mice [160]. IGFBP1 (insulin‐like growth factor binding protein) deletion increases FOXO3A protein levels, resulting in endothelial aging delay [161]. Single‐cell transcriptome sequencing results indicate that aging weakens FOXA2's activation of FOXO3A transcription, which is crucial for the aging of aortic and coronary vascular cells in monkeys, and knocking out FOXO3A mimics the vascular regeneration impairment caused by aging [162]. Another study indicates that endothelial aging leads to increased AKT activity, which suppresses FOXO3A and raises reactive oxygen species levels. This, in turn, activates the p53/p21 pathway, further promoting vascular endothelial aging [163].

#### 3.5.3 FOXO4

Research indicates that FOXO4 is crucial for the vitality of senescent cells. Interfering with FOXO4 using a peptide disrupts its interaction with p53, leading to p53 nuclear exclusion and the death of senescent cells, thereby delaying vascular natural aging in mice [164, 165]. This suggests that the FOXO4 peptide could be a promising gene‐engineered senolytic strategy.

### 3.6 Stem Cell and Its Secretion

Vascular aging is characterized by endothelial dysfunction, increased arterial stiffness, and a pro‐inflammatory state, all of which contribute to the deterioration of vascular health. Stem cells, particularly mesenchymal stem cells (MSCs) and induced pluripotent stem cells (iPSCs), have demonstrated significant potential in reversing these age‐related vascular changes [166, 167]. MSCs, derived from bone marrow, adipose tissue, or umbilical cord blood, possess the ability to differentiate into various cell types, including endothelial cells, which are crucial for maintaining vascular integrity [168].

The challenge in clinical applications of stem cell transplantation is the occurrence of rejection reactions. One solution is to extract effective anti‐aging components from stem cells. Extracellular vesicles serve as communication molecules between cells, carrying cellular components such as nucleic acids and proteins to regulate the functions of recipient cells. Induced pluripotent mesenchymal stem cell (iPSC) derived extracellular vesicles improved aging‐related vascular endothelial dysfunction, arterial stiffness, and hypertension by activating the SIRT1‐AMPKα‐eNOS pathway and suppressing MMPs and elastase [169]. Exosomes derived from mesenchymal stem cells contain high levels of miR‐146a, which can improve vascular function in aged mice by inhibiting Src phosphorylation and its downstream targets, VE‐cadherin and caveolin‐1 [170]. Neonatal umbilical cord‐derived MSC‐EVs (UC‐EVs) are rich in anti‐aging signals and mitigate aging‐related vascular phenotypes [171].

### 3.7 NO‐sGC‐cGMP Signaling

NO is a crucial regulatory molecule for vascular homeostasis, involved in processes such as vascular stiffness, inflammation, vasodilation, and angiogenesis. Its downregulation during aging leads to arterial remodeling and atherosclerosis. In endothelial cells, inflammatory factors stimulate endothelial nitric oxide synthase to produce NO, which penetrates deeper to reach smooth muscle cells and activate soluble guanylate cyclase (sGC). The activated sGC, through its C‐terminal catalytic domain, promotes the excessive secretion of the second messenger cGMP, leading to various cardiovascular responses such as vasodilation, leukocyte aggregation, and increased platelet activity. Insufficient cGMP secretion or reduced activity can result in endothelial dysfunction, vasoconstriction, vascular stiffness, adverse remodeling, and decreased renal and coronary blood flow [172]. Therefore, sGC activators play a crucial role in combating myocardial ischemia, improving left ventricular remodeling, alleviating heart failure, and inhibiting inflammation and fibrosis, as well as enhancing metabolism. Due to the significant limitations of NO donor drugs or inhaled NO in clinical treatment, enhancing sGC levels or inhibiting cGMP degradation has become a potential alternative.

In mice experiencing natural aging or aging induced by DNA repair gene defects (Ercc1^*∆/−*^), weakened NO‐sGC‐cGMP signaling has been observed in their blood vessels. This weakening is associated with an increase in PDE1, which reduces cGMP through hydrolysis [173, 174, 175]. The sGC activator and PDE1 inhibitor improved microvascular cutaneous perfusion and increased survival in progeroid Ercc1^∆/−^ mice [173, 176, 177]. Although PDE1 inhibition and sGC activation/stimulation appear to be promising strategies for combating vascular aging, most current research relies on mouse models of accelerated vascular aging. The optimal intervention window and administration timing for natural aging remain undetermined. Additionally, while the combined therapy of PDE1 inhibition and sGC regulation seems intriguing, it may lead to hypotension. Determining the appropriate dosage and administration method to avoid this issue could be beneficial for advancing vascular aging treatments [178].

## 4 The New Potential Methods for Vascular Aging Study

### 4.1 Aging Cell Living Imaging and Tracing

Accurate tracking and visualization of senescent cells is crucial for studying cellular aging mechanisms and evaluating anti‐aging drugs. Traditional assessments of cellular senescence rely on detecting markers such as cell cycle inhibitors (p16, p21), the SASP, and senescence‐associated β‐galactosidase (SA‐β‐Gal) activity. Most senescence marker detections are invasive, requiring tissue from sacrificed animals or fixed cells, making it difficult to evaluate anti‐aging drugs in living organisms. Therefore, tracking senescent cells, particularly in vivo, remains a significant challenge and an urgent issue in the field of aging research.

To address this issue, researchers have developed transgenic mice with aging reporter genes. The p16‐CreERT2‐tdTomato mouse model enables the analysis of p16 high cells in vivo characteristics at the single‐cell level. The tdTomato‐positive p16 high cells are detectable in all organs and increase with age, serving as an effective indicator of aging [179]. Using p16 reporter gene‐based mice, it was confirmed that liver vascular EC is among the earliest cell populations to undergo senescence in vivo. A significant presence of p16‐high vascular EC can be observed in mice as early as 10–12 months old, providing a valuable paradigm for determining the onset window of vascular aging [180, 181]. The p21‐3MR mouse model, which includes a p21 promoter‐driven module, enables the monitoring, imaging, and elimination of p21 high cells in vivo. This model serves as a powerful tool for studying p21 high cells to enhance our understanding of senescence biology [182]. The Glb1^+/m^–Glb1‐2A‐mCherry (GAC) reporter mice can be used to monitor cells with high expression of β‐galactosidase, as its signal level increases linearly with aging in mice aged 9–13 months. This model accurately identifies aging induced by bleomycin or inhibited by senolytics, providing a suitable animal model for screening anti‐aging drugs [183]. Recently, a chemical fluorescent probe, XZ1208, has been developed to detect SA‐β‐gal activity, in addition to genetically engineered mice. This probe can be rapidly recognized and cleaved by senescent cells, releasing near‐infrared fluorescent dye, allowing for highly specific labeling of senescent cells in various cell types and senescence models [184]. This method avoids the complex breeding steps required for transgenic mice and serves as a powerful tool for monitoring tissue and organ aging and screening anti‐aging drugs. However, a limitation of this approach is its inability to achieve cell‐specific labeling due to a lack of cell selectivity. If integrated into a highly selective vascular cell delivery system, it could become an efficient tool for studying vascular aging through specific labeling.

These fluorescently labeled tracers enable comprehensive monitoring of vascular aging, facilitating the study of its spatiotemporal dynamics and the in vivo screening of anti‐aging drugs. Additionally, they allow for the isolation of senescent vascular cells via flow cytometry for multi‐omics sequencing, which identifies changes in genes, proteins, metabolism, and epigenetics, thereby advancing our understanding of the mechanisms underlying vascular aging [185].

In addition to studying natural vascular aging, it is intriguing to explore the role of vascular senescence in other vascular diseases, particularly in vascular development, arteriogenesis, capillary arterialization, and vascular injury. A notable example is the formation of collateral circulation due to coronary artery stenosis, which rapidly regresses after PCI. Whether this process involves coronary artery senescence or is merely due to reduced eNOS levels from altered mechanical stress is a significant topic of interest.

### 4.2 CRISPR Library Screen

The CRISPR library screening technique is an unbiased, genome‐wide screening tool that identifies key candidate molecules from thousands of genes by employing various screening strategies [186]. It is a powerful method for rapidly identifying novel functional genes. Although it has not yet been applied to vascular aging research, it has been successfully used in identifying aging‐related genes in stem cells, notably recognizing RPL22, H2AZ1, SOX5, XPO7, and KAT7 as crucial regulatory molecules in stem cell aging [187, 188, 189, 190, 191]. Infecting vascular cells in vitro with GeCKO or GeCKI lentivirus and then sequencing the dominant cell subpopulations that proliferate under senescence resistance is a feasible approach to identifying genes that may delay or reverse vascular cell aging. Furthermore, senolytics can be used in conjunction with CRISPR library‐infected vascular cells to target and eliminate cells that age rapidly due to gene deletion or overexpression. Sequencing the dead cells can then identify key genes responsible for cellular aging. Similar death screening strategies have been proven reliable in previous studies [192, 193].

Combining CRISPR libraries with single‐cell sequencing (Perturb‐seq) for in vivo screening of key genes involved in vascular aging is a viable strategy [194]. This approach enables parallel screening of multiple genes in mice or vascular organoids to identify candidate genes that significantly contribute to vascular aging and to explore potential mechanisms. This strategy is crucial because not all aging‐related genes equally affect vascular aging. It helps identify stronger candidate genes, providing evidence for potential targets for effective vascular aging interventions.

## 5 Challenges and Directions of Vascular Aging Research

Despite extensive research on vascular aging over the past few decades, many complex issues remain unresolved. One of the most critical challenges is accurately assessing the physiological age of blood vessels. In preclinical studies, researchers often use SA‐β‐gal staining or measure the expression of senescence markers such as p16INK4a, p21CIP1, LaminB1, and γH2AX to indicate the degree of vascular aging [34, 127]. However, these methods are mostly semi‐quantitative and require a control group for comparison. In clinical practice, semi‐quantitative methods are not acceptable due to the lack of appropriate control groups. It is difficult to determine whose vascular age aligns with their physiological age, and obtaining vascular tissue for staining or molecular biology testing is challenging.

Therefore, there is an urgent need for circulating biomarkers or non‐invasive methods to assess vascular age. Currently, PWV and FMD are commonly used non‐invasive methods to evaluate vascular function [195, 196, 197]. These methods effectively assess vascular stiffness and endothelial function, particularly in older patients with increased vascular stiffness and reduced dilation function. However, they are less effective in identifying individuals whose vascular age may be younger than their physiological age. Additionally, these methods can only indicate whether the vascular age is older than the physiological age but cannot quantify the degree of aging. Regarding circulating biomarkers, there are currently no sensitive markers available for evaluating vascular aging. This highlights the need for further research to develop reliable biomarkers and non‐invasive techniques for accurately assessing vascular age.

One of the challenges in vascular aging research is vascular heterogeneity. Although blood vessels form an interconnected network within the human body, arteries, veins, and capillaries exhibit significant differences in their aging characteristics due to their distinct compositions [198, 199, 200]. Large arteries primarily show thickening of the vessel wall and reduced elasticity. In contrast, veins exhibit an increased risk of thrombosis, while capillaries demonstrate degradation of the vascular network. Additionally, the functions of blood vessels vary across different organs. For instance, the barrier and permeability functions of blood vessels are crucial for the brain, kidneys, lungs, and liver, but may be less critical for other organs [115, 201, 202, 203]. The degradation of the vascular bed can lead to tissue ischemia and hypoxia, with myocardial cells and neurons being highly sensitive to such conditions, whereas the kidneys have better tolerance to hypoxia [204, 205]. These variations in aging manifestations and outcomes pose challenges for studying vascular aging and developing interventions. Research focused on the vascular aging of a single organ may be limited, as the mechanisms and targets identified may not apply to all blood vessels. A typical example is the deletion of FOXO3A, which leads to aging in HAEC and hESC‐derived heVEC, but does not affect HUVEC, indicating differences in the aging mechanisms of various endothelial cells [162]. Therefore, identifying common aging mechanisms across different types of blood vessels and even across species may be necessary. This approach could help overcome the limitations of studying vascular aging in a single organ.

Another complex issue in clinical research is determining the outcomes of vascular aging. Currently, there are very few clinical studies focused on vascular aging. One significant reason for this is the considerable controversy surrounding the endpoints for observing vascular aging. The boundaries between increased arterial stiffness, atherosclerosis, and the risk of venous thrombosis due to aging and deep vein thrombosis in the lower limbs are very blurred. It is challenging to distinguish whether the primary drivers of increased arterial stiffness and thrombosis are traditional cardiovascular risk factors or aging itself. Therefore, a comprehensive evaluation strategy is needed to indicate the outcomes of vascular aging.

Existing aging clocks based on circulating multi‐omics have been established, but they predominantly use data from the UK Biobank or populations with Western diets [206, 207]. Dietary differences between countries may lead to variations in the abundance of circulating proteins. Although studies have shown that diets like the Mediterranean diet have a stronger protective effect against vascular aging, the goal of extending lifespan does not show significant differences between Eastern developed countries such as Japan and Singapore, and developing countries like China, compared to developed Western countries with predominantly Mediterranean diets. This suggests that dietary differences may not be the core factor determining lifespan. Furthermore, while lifespan or age at death is related to vascular aging, vascular aging is not necessarily the primary determinant of lifespan. Thus, using all‐cause mortality or age at death as endpoints for observing vascular aging is unreliable. These findings indicate a significant gap between research results obtained from mice, worms, and monkeys and their clinical translation and application, possibly due to genomic differences among species. A promising solution could be human vascular organoids, which possess a human genome and allow for the testing of various drugs and gene modification techniques in vitro. Although vascular organoid models have recently been developed, their long aging half‐life limits their use in studying natural aging, despite being much shorter than that of mice [208, 209, 210]. Nonetheless, they are undoubtedly well‐suited for research on inducible human vascular aging.

In conclusion, as the global population continues to age, the importance of understanding and addressing vascular aging becomes increasingly paramount. Future research should focus on elucidating the complex interplay between genetic, epigenetic, and environmental factors in vascular aging, as well as developing personalized therapeutic strategies to improve vascular health and prevent age‐related cardiovascular diseases.

## Author Contributions

Conceptualization, methodology, writing – original draft: W.‐Z.W. and X.W. Investigation, visualization: F.‐F.W. Funding acquisition, project administration, supervision: Y.‐P.B., W.‐Z.W. and X.‐T.Y. Writing – review and editing: all authors.

## Conflicts of Interest

The authors declare no conflicts of interest.
